# Automated insulin delivery in war refugee children with type 1 diabetes: a 3-year follow-up

**DOI:** 10.3389/fendo.2026.1901242

**Published:** 2026-07-24

**Authors:** Vit Neuman, Lukas Plachy, Stepanka Pruhova, Stanislava Kolouskova, Barbora Obermannova, Lenka Drnkova, Shenali Anne Amaratunga, Petra Konecna, Jana Vyzralkova, Petra Venhacova, Renata Pomahacova, Petra Paterova, Lucie Stichova, Jaroslav Skvor, Kamila Kocourkova, Martina Romanova, Jan Vosahlo, Jiří Strnadel, Kristina Polockova, Jan Knizek, David Neumann, Matvei Slavenko, Marketa Pavlikova, Zdenek Sumnik

**Affiliations:** 1 Department of Pediatrics, 2nd Faculty of Medicine, Charles University and Motol and Homolka University Hospital, Prague, Czechia; 2 Department of Pediatrics, University Hospital Brno, Brno, Czechia; 3 Department of Pediatrics, University Hospital Olomouc, Olomouc, Czechia; 4 Department of Pediatrics, University Hospital Pilsen, Pilsen, Czechia; 5 Department of Pediatrics, Masaryk Hospital, Usti nad Labem, Czechia; 6 Department of Pediatrics, Ceske Budejovice Hospital, Ceske Budejovice, Czechia; 7 Department of Pediatrics, 3rd Faculty of Medicine, Charles University and Kralovske Vinohrady University Hospital, Prague, Czechia; 8 Department of Pediatrics, University Hospital Ostrava, Ostrava, Czechia; 9 Department of Pediatrics, Karvina Hospital, Karvina, Czechia; 10 Department of Pediatrics, University Hospital Hradec Kralove, Hradec, Kralove, Czechia; 11 Department of Pediatrics, Trutnov Hospital, Trutnov, Czechia; 12 Department of Probability and Mathematical Statistics, Faculty of Mathematics and Physics, Charles University, Prague, Czechia

**Keywords:** automated insulin delivery (AID), continuous glucose monitor (CGM), pediatrics, type 1 diabetes (T1D), war refugees

## Abstract

**Objective:**

Following the war in Ukraine, Europe received a large influx of war refugees, including children with type 1 diabetes (CwD). The aim of this study was to describe their diabetes control during the first three years.

**Research design and methods:**

A total of 143 (70 M, 73 F) CwD were recruited into this longitudinal observational study between March 2022 and June 2025 at 9 tertiary pediatric diabetes centers. The following data were collected yearly: treatment type, CGM use, anthropometrics, HbA1c, and CGM metrics. Statistical inference was performed using linear mixed models adjusting for age, sex, age at T1D onset, and therapy type. Additionally, the participants were compared to matched controls from a nationwide pediatric diabetes registry.

**Results:**

The median HbA1c decreased significantly from 58.0 mmol/mol, 7.5% (IQR 49.0-73.0, 6.6–8.8%) to 52 mmol/mol, 6.9% (IQR 48–64 mmol/mol, 6.5–8.0%) over 3 years (P<0.001). We observed a significant increase in CGM use (46.4% to 95.5%) and insulin pump use, from 8.0% (no AID systems) to 63.6% (only AID systems) (both P<0.001). The trend in HbA1c decline depended on the therapy type, the estimated effect of AID initiation was −5.39 mmol/mol (95% CI −10.12 to −0.65 mmol/mol) (P = 0.003). The mean time in range (TIR) of CwD who initiated AID was not significantly different from that of matched non-migrant controls (73.4% vs. 71.0%, P = 0.420), while those using insulin pens had a lower TIR than controls (51.8% vs. 62.9%, P = 0.003).

**Conclusions:**

We observed a significant improvement in T1D control among refugee CwD, emphasizing the role of AID, regardless of socioeconomic background.

## Highlights

- Czechia received a large number of refugees from Ukraine, including children with type 1 diabetes (CwD). These children were followed for 3 years to identify the impact of treatment changes on T1D control.
- Are technologies (mainly CGM and AID systems) effective in improving T1D control in a disadvantaged population? Can refugee CwD reach similar T1D control as non-refugee CwD?
- HbA1c decreased significantly during the follow-up period, with a more pronounced decline in CwD who initiated CGM and AID. CwD who initiated AID therapy reached similar T1D control as their well-matched non-refugee peers, while those who stayed on a multiple daily injection regimen had worse outcomes.
- Our data emphasize the role of CGM and AID in improving T1D control in all CwD, regardless of their socioeconomic background.

## Introduction

Despite significant improvements in the metabolic control of children with type 1 diabetes (CwD) in recent years (1), there are still significant differences around the world that can be partially attributed to the unevenness in access to diabetes technology, particularly continuous glucose monitoring (CGM) and automated insulin delivery systems (AID) (2). Even within one country, the use of modern diabetes technology is highly dependent on the socioeconomic status of the family (3) and often translates into an increased risk of acute or chronic complications and a lower quality of life for CwD from an underprivileged background (4–6). These associations are particularly apparent in CwD from migrant populations (7, 8).

After the outbreak of war in Ukraine, Central Europe has become one of the primary target locations for war refugees (9). Due to laws prohibiting adult men from crossing the borders, women and children (including CwD) were disproportionately included among them. Upon arriving in their host countries, many sought out larger diabetes centers for further care (10). We have previously demonstrated that despite many being lost to follow-up, those who stayed greatly benefited from the introduction of CGM in their first year (10). Another interesting finding was the increase in BMI among these CwD, which could be attributed to more liberal eating habits (11). A registry-based observational study from the DPV (including Germany, Austria, Switzerland, and Luxembourg) further emphasized the role of CGM in migrant populations, as the group of Syrian/Afghan origin had the lowest CGM uptake and the highest HbA1c (12). However, neither of these studies focused on the role of AID systems, as this technology was recently developed, and the uptake rate was quite low.

The aim of this study was to describe the changes in technology uptake and its association with diabetes control and the anthropometric features of Ukrainian war refugee CwD during the first 3 years of their stay in Czechia, comparing them with matched controls from a non-migrant background.

## Methods

### Study population characteristics

The current study is an extension of a previous study, with the methods published elsewhere (10). Briefly, all children and adolescents under 19 years of age with type 1 diabetes who were diagnosed in Ukraine and registered at one of the participating secondary or tertiary centers for diabetes care in Czechia (9 in total, all of which care for over 100 CwD in Czechia) between March 2022 and June 2025 were offered the opportunity to participate in this observational study. During the first visit at their registration center, baseline demographic, anthropometric, and diabetes-related information, including pre-existing diabetes complications, was obtained (detailed in **Table 1**). A bilingual informed consent in Ukrainian and Czech was obtained from the parents/caregivers of each participant. Data from all consenting participants were entered in yearly intervals for 3 years or until June 2025, whichever came first. The study was approved by the Ethics Committee of Motol University Hospital, Prague, Czechia.

**Table 1:** Baseline characteristics of the study participants.

Characteristic	Number of subjects	Study subjects	
Basic demography			
Sex	143	F=73, M = 70	
Age (years)	132	12.8 [8.7; 15.0], 11.8 (3.8)	
Duration of diabetes (years)	118	3.0 [1.5; 6.0], 4.3 (3.7)	
Anthropometric data			
Body weight SDS	118	−0.13 [−0.79; 0.56], −0.02 (1.04)	
Body height SDS	118	−0.36 [−0.98; 0.50], −0.33 (1.17)	
BMI SDS	118	0.03 [−0.57; 0.67], 0.17 (1.03)	
Diabetes-related characteristics			
Treatment modality	138	MDI=127, CSII = 11	
CGM status	138	Yes=64, No=74	
HbA1c			
mmol/mol	124	58.0 (49, 73), 63.5 (21.5)	
DCCT %	124	7.5 [6.6; 8.8], 8.0 (4.1)	
Time in CGM range (%)			
TIR (3.9–10 mmol/L), (70–180 mg/dL)	32	61.0 [55.0; 75.3], 61.7 (19.3)	
TBR1 (3.0, 3.8 mmol/L), (54–70 mg/dL)	27	4.0 [1.0; 8.0], 4.4 (4.2)	
TBR2 (<3.0 mmol/L), (<54 mg/dL)	27	0.0[0.0; 0.5], 1.0(2.5)	
TAR1 (10.1, 13.9 mmol/L), (180–250 mg/dL)	25	19.0 [13.0; 29.0], 21.0 (10.8)	
TAR2 (>13.9 mmol/L), (>250 mg/dL)	25	7.0 [2.0; 10.0], 11.4 (17.2)	
Average glycemia			
mmol/L	25	8.1 [7.2; 10.4], 9.3 (3.0)	
mg/dL	25	146.0 [129.8; 187.4], 167.6 (54.1)	
Coefficient of variation (%)	13	40.5 [36.6; 40.6], 41.1 (10.5)	
Associated diseases and complications		Yes	No
Autoimmune thyroiditis	116	20	96
Coeliac disease	113	5	108
Microalbuminuria	94	4	90
Retinopathy	66	0	66

BMI, body mass index, CGM, continuous glucose monitoring, CSII, continuous subcutaneous insulin infusion, DCCT, Diabetes Control and Complications Trial, F, female participant, HbA1c, glycosylated hemoglobin, M, male participant, MDI, multiple daily injections, Q1 and Q3, first and third quartiles, SDS, standard deviation score, TIR, time in the target range, TBR1, time in level 1 hypoglycemia, TBR2, time in level 2 hypoglycemia, TAR1, time in level 1 hyperglycemia, TAR2, time in level 2 hyperglycemia.

Depending on the characteristic, the data are reported as either counts in categories or as medians [Q1; Q3] and means (SD).

### Clinical and laboratory data

At the initial contact of the CwD with the participating center, baseline HbA1c and screening for diabetes comorbidities and complications were performed. The baseline characteristics are detailed in **Table 1**. During each study visit (baseline visit, M12, M24, and M36), changes in glucose monitoring or treatment modalities were noted. CGM-derived data were taken from the 14 days preceding each study visit and downloaded using specialized software (LibreView, Diasend, Glooko, or CareLink). Standard CGM metrics (13) were used for the analysis:

- time in:
  ▪ target range (TIR, between 3.9 and 10.0 mmol/L, 70–180 mg/dL),
  ▪ level 1 hypoglycemia (TBR1, between 3.0 and 3.8 mmol/L, 54–70 mg/dL),
  ▪ level 2 hypoglycemia (TBR2, below 3.0 mmol/L, <54 mg/dL),
  ▪ level 1 hyperglycemia (TAR1, between 10.1 and 13.9 mmol/L, 180–250 mg/dL),
  ▪ level 2 hyperglycemia (TAR2, above 13.9 mmol/L, >250 mg/dL),
- average glycemia (AG)
- coefficient of variation (CV)
- standard deviation of glycemia (SD)

Data on rates of diabetic ketoacidosis (DKA) and severe hypoglycemia (SH) were also collected during each visit. The CGM data were collected during the baseline visit from CwD who had a CGM device from Ukraine. Therapy type was divided into the following categories: multiple daily injections (MDI), continuous subcutaneous insulin infusion without automated insulin delivery (CSII), and automated insulin delivery (AID). All subjects who changed therapy types throughout the study were initiated on AID. All subjects initiated on AID were within the age indications of the respective AID systems. Weight and height were measured, and body mass index (BMI) was calculated, with standard deviation score values calculated from the population-based data (14). HbA1c was measured in certified local laboratories during each of the study visits. The data were anonymized at the level of each center and then transferred to the coordinating center (Motol University Hospital, Prague) for data analysis.

### Statistical analysis

To assess CGM and AID uptake while accounting for intrapatient dependencies, we used McNemar’s test. To perform statistical analysis of longitudinal continuous outcomes, we employed linear mixed models with a random intercept at the patient level. For the first set of models (crude analysis), the regressors included only time point (categorical, Baseline vs. M12 vs. M24 vs. M36). For the second set of models (covariates adjusted), regressors included therapy type (binary: MDI vs. CSII/AID), time point (categorical, Baseline vs. M12 vs. M24 vs. M36), date of first contact (or enrollment to the study, continuous), age at first contact (continuous), age at diabetes onset (continuous), and participant sex.

For the matched analysis, we included all patients who reached the M36 time point. Controls were drawn from the CENDA registry (15) (year 2025), restricted to patients with Czech nationality who were treated at large tertiary centers. Matching was performed in a one-to-many fashion: each study participant (case) was matched to one or more controls, while each control was assigned to at most one case. The matching variables included sex, therapy type, age, and age at diabetes onset. For three cases with missing data on age at diabetes onset and one case with missing therapy type data, matching was performed individually, excluding the missing data and using stricter matching on participant age.

To perform statistical inference on the matched dataset, we employed linear mixed models with a random intercept for each matched set (case). The regressors included patient status (case vs. control), therapy type, and their interaction. The median (IQR) was used for the presentation of HbA1c, anthropometric, and insulin dose data, while CGM values were presented as the mean (SD) to allow for the standard depiction of ambulatory glucose profiles.

The statistical analysis was conducted using the R programming language, v.4.5.0 (16). Linear mixed models were fitted using the “lme4” package. Statistical inference for the fixed coefficients of the mixed models was performed using Wald-type t- and F-procedures with the Satterthwaite approach for correcting degrees of freedom using the “lmerTest” package (17). All models were pre-specified, and no data-driven model-building procedures were employed. All tests were conducted at the α=0.05 significance level; confidence intervals were constructed with 95% coverage probability. No multiple comparison corrections were applied.

## Results

A total of 143 CwD (73 girls, 70 boys) were enrolled in the study between March 2022 and June 2025. Their baseline characteristics are shown in **Table 1**. Briefly, the vast majority (88.8%) of the subjects were using multiple daily injections (MDI), with the rest using CSII (7.7%) or having missing data (3.5%). No AID use was noted at the baseline visit. Fewer than half of the CwD (44.8%) were using CGM at the time of their arrival to Czechia. The rates of acute complications were very low throughout the study, with only two severe hypoglycemia events and two diabetic ketoacidosis episodes noted during the 3-year follow-up period. We noted a high attrition rate throughout the study, particularly in some participating centers and especially in the first year of follow-up (**Supplementary Table 1**). The attrition rate per center is detailed in **Supplementary Table 2**. The group of participants did not completed the entire follow-up was significantly older (P = 0.01) with a higher age of T1D onset (P<0.01) than those who were lost to follow-up or who did not reach the final study visit (**Supplementary Table 3****).**

The HbA1c decreased significantly throughout the course of the study with a drop from a median of 58 mmol/mol, 7.5% (IQR 49–73 mmol/mol, 6.6–8.8%) to 52 mmol/mol, 6.9% (IQR 48–64 mmol/mol, 6.5–8.0%), P<0.001 for the overall effect of time in the mixed model, both crude and covariate-adjusted. We observed a significant increase in CGM use, rising from 44.8% at baseline to 95.5% after 36 months (P<0.001, McNemar’s test) (**Supplementary Table 4**). Similarly, CSII use increased from 7.7% at baseline (with no AID systems) to 63.6% (all using AID systems) at 36 months (P<0.001, McNemar’s test).

During the follow-up, the mean TIR of the subjects increased from 61.7% (SD 19.3%) to 66.2% (SD 17.0%) (P = 0.04 for time point in crude model, P = 0.95 for covariate-adjusted effect). The CGM parameters are detailed further in **Supplementary Figure 6** and **Supplementary Table 7**. Throughout the study, we observed significant increases in the median body weight SD from −0.13 (IQR −0.79 to 0.56) to 0.14 (IQR −0.45 to 0.98) (P<0.001 for time point in both crude and covariate-adjusted models) and in the BMI SD from 0.03 (IQR −0.57 to 0.67) to 0.33 (IQR −0.39 to 1.12) (P<0.001 for time point in both models). The relative insulin dose did not change significantly during this time, with a median of 0.80 IU/kg/day (IQR 0.61 to 0.97 IU/kg/day) at baseline vs. 0.87 IU/kg/day (IQR 0.77 to 1.05 IU/kg/day) at 36 months (for time point effect, crude P = 0.18 and covariate-adjusted P = 0.17).

**Figure 1:**
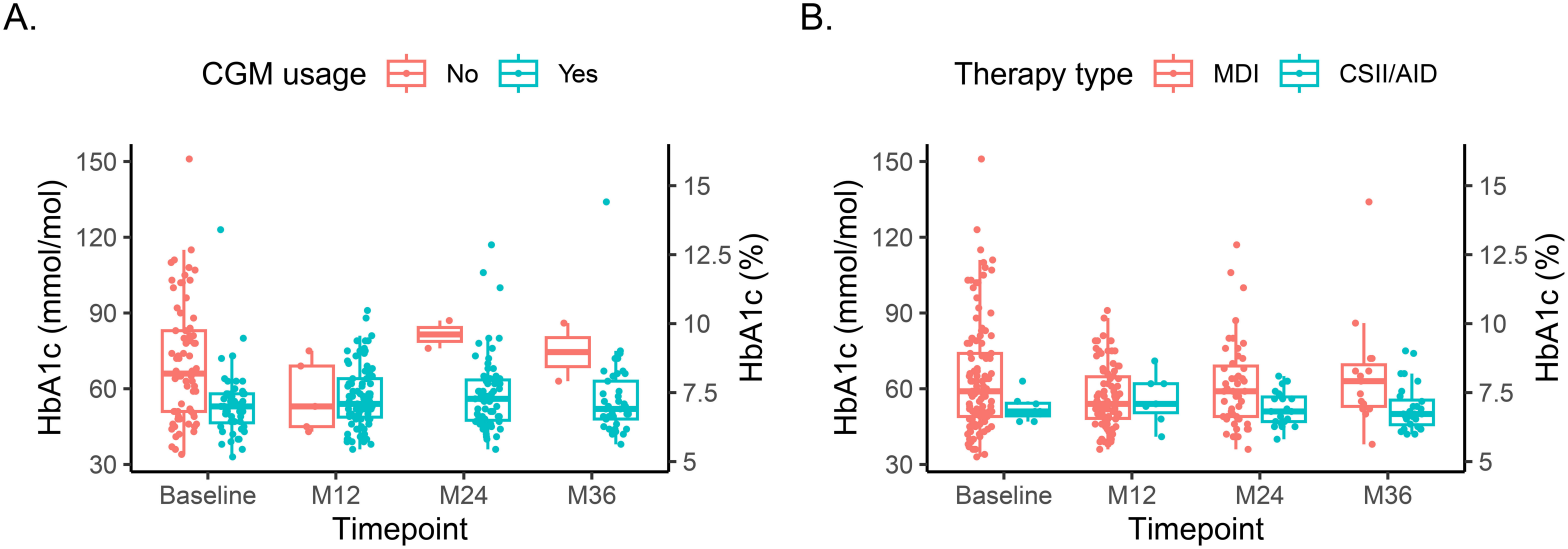
**(A)** HbA1c values in participants according to CGM status. The estimated effect of CGM initiation was −9.33 mmol/mol (95% CI −13.71 to −4.96 mmol/mol) (P<0.001). **(B)** HbA1c values in participants according to their MDI vs. CSII/AID status. The estimated effect of AID initiation was −5.39 mmol/mol (95% CI −10.12 to −0.56 mmol/mol) (P = 0.026). P-values correspond to tests of the coefficients in linear mixed models adjusted for covariates.

**Figure 2:**
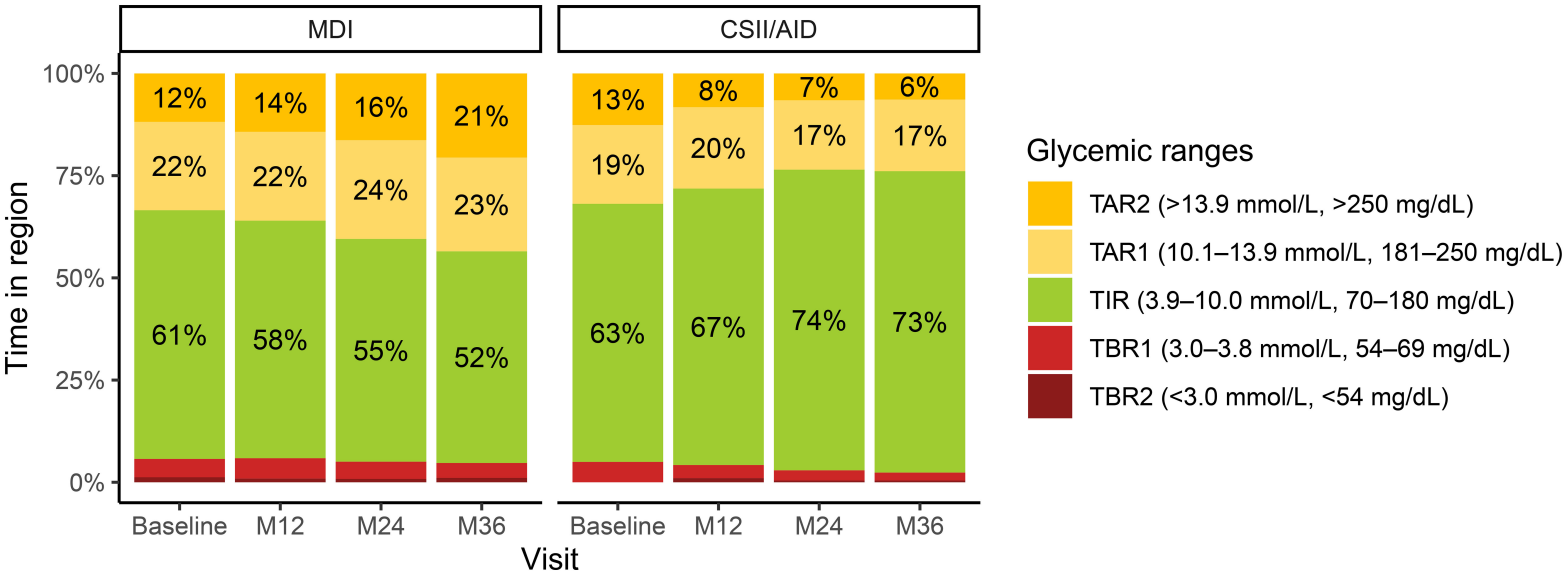
Means of times in glycemic ranges of the participants according to therapy type (MDI vs. CSII/AID). Therapy with AID was associated with an estimated average 16.69% increase in TIR (95% CI 11.26 to 22.12, covariate-adjusted P<0.001).

HbA1c did not differ significantly between male and female patients (covariate-adjusted P = 0.72). The initiation of CGM led to a significant decrease in HbA1c, with an estimated effect of −9.33 mmol/mol (95% CI −13.71 to −4.96 mmol/mol) (P<0.001) (**Figure 1A**). Similarly, the introduction of AID had a significant effect on HbA1c levels, with an estimated effect of −5.39 mmol/mol (95% CI −10.12 to −0.56 mmol/mol) (P = 0.026) (**Figure 1B**). Therapy with AID was associated with an estimated 16.69% increase in TIR (95% CI 11.26 to 22.12, covariate-adjusted P<0.001), a −1.56% decrease in TBR1 (95%CI −2.83 to −0.3, covariate-adjusted P = 0.016), and a 0.54% decrease in TBR2 (95%CI −1.18 to 0.1, covariate-adjusted P = 0.097). Similarly, TAR1 decreased by −6.45% (95%CI −9.73 to −3.16, covariate-adjusted P<0.001) and TAR2 by –9.71% (95%CI −14.22 to −5.2, covariate-adjusted P<0.001) (**Figure 2**). The estimated effect of therapy type on other CGM parameters is shown in **Supplementary Table 8**. We did not observe a significant effect for therapy with AID on BW SD −0.1 (95% CI −0.34 to 0.14) (covariate-adjusted P = 0.39), BMI SD −0.16 (95% CI −0.44 to 0.11) (covariate-adjusted P = 0.25) or relative insulin dose −0.06 IU/kg/day (95% CI −0.17 to 0.06 IU/kg/day) (covariate-adjusted P = 0.32).

**Figure 3:**
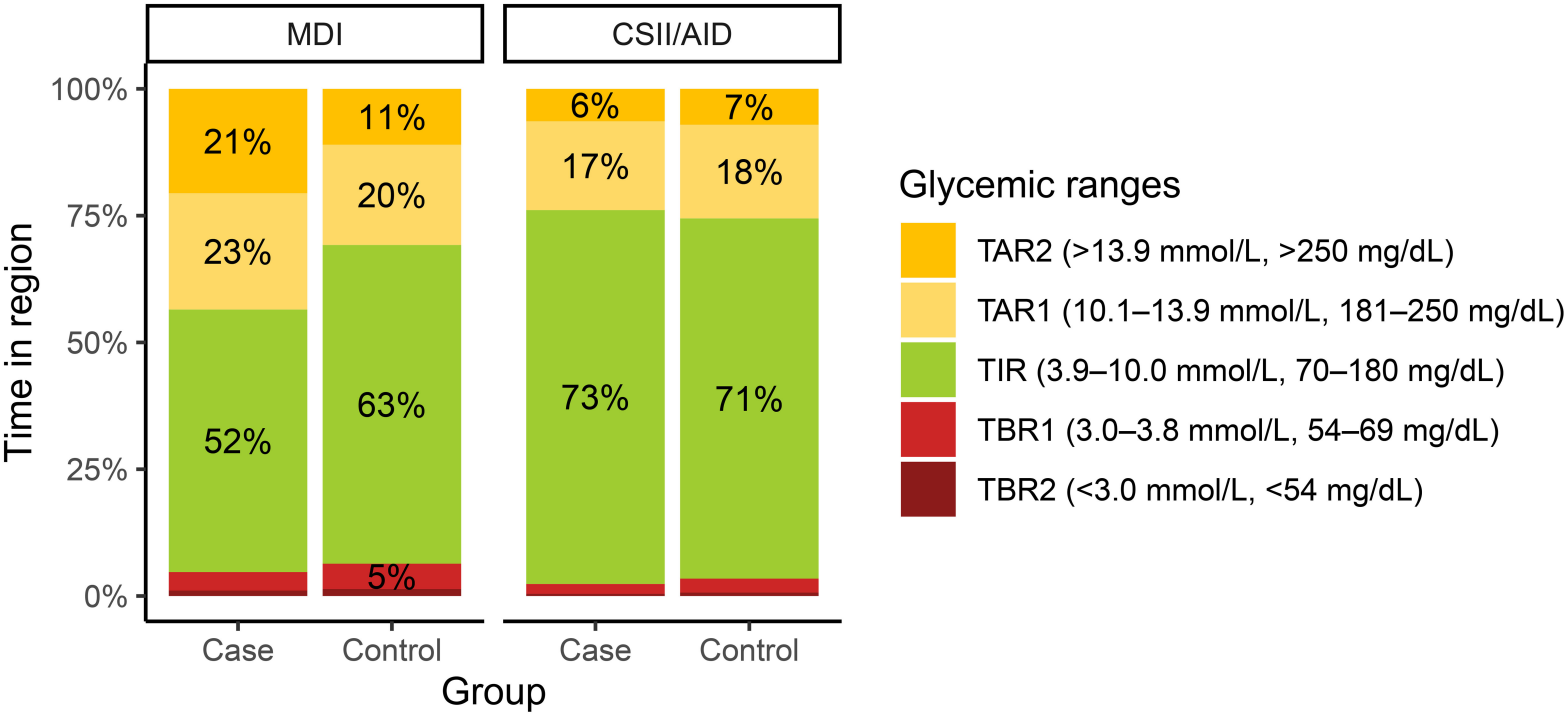
Mean times in glycemic ranges of the study participants (case) and their matched controls from the Czech National Diabetes Registry (control) at 3 years according to the type of therapy (MDI vs. CSII/AID). While the differences were insignificant in the CSII/AID group, there were significant differences in the MDI group with lower TIR (−10.9%, P = 0.003) and higher TAR2 (+10.0%, P = 0.002) in cases.

At 3-years, CwD from Ukraine (N = 45) achieved similar HbA1c levels as their matched peers from the Czech National Pediatric Diabetes Registry (N = 91) with HbA1c of 52 mmol/mol, 6.9% (IQR 48 to 64 mmol/mol, 6.5 to 8.0%) vs. 53 mmol/mol, 7.0% (48 to 57 mmol/mol, 6.5 to 7.4%) (P = 0.1, 95% CI −7.75 to 0.64). When stratified by therapy type, only CwD on AID achieved similar results to their peers from a non-migrant background (50 vs. 52 mmol/mol, 6.7 vs. 6.9%, P = 0.97, 95% CI −5.03 to 5.20), while those on MDI had significantly higher HbA1c than the matched controls (63 vs. 53 mmol/mol, 7.9 vs. 7.0%, P<0.001, 95% CI −17.7 to −4.4). Similarly, the CGM parameters were not significantly different between the unstratified groups (**Supplementary Figure 9**), but when stratified by therapy type, only the values in the AID group remained non-significant. In the MDI group, CwD from Ukraine had a significantly lower TIR of 10.9% (95%CI 3.7 to 18.1, P = 0.003) and a higher TAR2 by 10.0% (95%CI 3.8 to 16.1, P = 0.002). CwD from Ukraine had a 1.5-percentage-point lower TBR1 than matched controls, but the difference was not statistically significant (95% CI 0.0 to 3.0, P = 0.05). These data are shown in **Figure 3**.

## Discussion

Our data indicate that it is possible to achieve favorable glycemic outcomes in war refugee CwD. Equal access to diabetes technology, especially CGM and AID systems, appears to be the key feature in this respect. Over 3 years, migrant CwD who adopted diabetes technology were able to reach HbA1c levels similar to those of their non-migrant counterparts, achieving similarly low levels of acute complications.

The increasing number of refugees worldwide (18) poses a significant challenge to healthcare systems. Children and adolescents with diabetes from a migrant background often do not meet therapeutic targets and have higher levels of acute and chronic complications even in high-income countries (7, 19), which translates to a higher economic burden (20, 21). Additionally, the quality of life was shown to be poorer for these CwD and their parents (8). Our data suggest that the early adoption of diabetes technologies can be particularly useful in addressing some of these issues.

The current study provides a prolonged follow-up of a previously published cohort (10). In the previous study, we had noted that CGM was a significant factor in reducing HbA1c levels in war refugee CwD shortly after their arrival in the host country, although it was not sufficient for achieving optimal outcomes. The current, expanded dataset, with prolonged follow-up, allowed us to draw further inferences with regard to AID therapy, which equalized the outcomes of migrant CwD with those of non-migrant CwD, as opposed to those who remained on MDI. On the other hand, Auzenneau et al. have shown that CwD from Ukraine had a higher tendency to adopt diabetes technology than CwD from other migrant backgrounds, suggesting that this solution might not apply to all migrant populations (12).

We have observed an increase in BMI SD in Ukrainian war refugee CwD, which was most apparent during the first year of their stay in Czechia. Because the relative dose of insulin did not change, we speculate that this was due to a more liberal approach toward food in Czechia rather than due to underdosing insulin, as previously discussed (10). The fact that this change occurred in the first year and BMI remained stable afterward suggests that the increase was not linked to AID use. Other factors may have influenced these results, including an improvement in living conditions after migration. On the other hand, the normal baseline values of body height and BMI SDS at baseline in refugee CwD likely rule out unfavorable T1D control as the reason for the BMI increase. The slightly increased BMI is in line with findings that CwD often have higher values than children without T1D (22), a trend also described in Czechia (15).

Our study has several strengths. First, its longitudinal nature and a rather long follow-up period allowed us to observe the trends in diabetes control and therapy and assess their durability. Second, the multi-center design and the use of a nationwide registry for the matched analysis increase the generalizability of our findings. Additionally, the insurance policy in Czechia allowed us to initiate diabetes technology for all CwD with a war-refugee background, partly limiting selection bias.

However, there are several limitations to our study. First, its observational nature limited our ability to draw any causal conclusions. Second, the reliability and generalizability of our findings were limited due to the high rate of loss to follow-up (probably due to remigration or return of the CwD), which may have increased selection bias. However, we believe that the number of CwD who attended visits was sufficient to justify our conclusions. Also, despite the generally low HbA1c values, several outliers with very high values are present in our dataset, supporting the idea that not only highly motivated CwD were followed at the participating centers. Another limitation is the lack of data on the socioeconomic status of families, such as parental level of education. These were not collected due to possible conflicts with data protection regulations in the EU. Nevertheless, we believe that these factors may initially have a rather minor effect, as even parents/caregivers of refugee CwD with a higher education would not be able to perform their previous occupations in Czechia until passing language and proficiency tests.

In conclusion, we observed a significant improvement in T1D control among war refugee CwD from Ukraine during the first 3 years of their stay in Czechia. Our data emphasize the crucial role of adopting AID systems in improving T1D control in all CwD, regardless of their socioeconomic background.

## Data Availability

The raw data supporting the conclusions of this article will be made available by the authors, without undue reservation.
