# Supplementary material for: Automated insulin delivery in war refugee children with type 1 diabetes: a 3-year follow-up
Source: Front Endocrinol (Lausanne). 2026 Jul 24;17:1901242. doi: 10.3389/fendo.2026.1901242 (PMC13447148; doi:10.3389/fendo.2026.1901242)
Supplement: Supplementary file 1 [file SupplementaryFile1.docx]

**ESM Table 1** – Visit status of the participants at each visit (N=143).

|  | **Baseline visit** | **M12** | **M24** | **M36** |
| --- | --- | --- | --- | --- |
| **Attended** | 143 (100%) | 91 (63.6%) | 71 (49.7%) | 45 (31.5%) |
| **Missed** | 0 (0%) | 7 (4.9%) | 0 (0%) | 0 (0%) |
| **Timepoint not reached** | 0 (0%) | 3 (2.1%) | 10 (7%) | 32 (22.4%) |
| **Lost to follow-up** | 0 (0%) | 42 (29.4%) | 62 (43.4%) | 66 (46.2%) |

M12 – month 12, M24 – month 24, M36 – month 36

**ESM Table 2** – Attrition of study subjects per visit for each participating center and the percentage of these from the baseline visit.

| **Visit** | **Medical center** | | | | | | | | |
| --- | --- | --- | --- | --- | --- | --- | --- | --- | --- |
|  | **UHM** | **UHB** | **UHKV** | **UHP** | **UHHK** | **HUNL** | **UHO** | **UHOL** | **HCB** |
| **Baseline** | 0 (0%) | 0 (0%) | 0 (0%) | 0 (0%) | 0 (0%) | 0 (0%) | 0 (0%) | 0 (0%) | 0 (0%) |
| **M12** | 14 (26.4%) | 7 (33.3%) | 6 (31.6%) | 7 (58.3%) | 5 (55.6%) | 2 (25.0%) | 9 (60.0%) | 2 (100%) | 0 (0%) |
| **M24** | 23 (43.4%) | 11 (52.4%) | 4 (21.1%) | 7 (58.3%) | 6 (66.7%) | 3 (37.5%) | 13 (86.6%) | 2 (100%) | 3 (75.0%) |
| **M36** | 27 (50.9%) | 16 (76.2%) | 12 (63.2%) | 12 (100%) | 7 (77.8%) | 5 (62.5%) | 13 (86.6%) | 2 (100%) | 4 (100%) |

UHM – University Hospital Motol, UHB – University Hospital Brno, UHKV – University Hospital Kralovske Vinohrady, UHP – University Hospital Pilsen, UHHK – University Hospital Hradec Kralove, HUNL – Hospital Usti nad Labem, UHO – University Hospital Ostrava, UHOL – University Hospital Olomouc, HCB – Hospital Ceske Budejovice, M12 – month 12, M24 – month 24, M36 – month 36

**ESM Table 3** – Baseline characteristics comparison among participants who completed the study follow-up (Completers, N=45) and those lost to follow-up or nor reaching the final timepoint (M36) (Non-completers, N=98). The non-completers were significantly older (P=0.009) with higher age at T1D onset (P=0.002). Data are shown as mean (SD).

| **Characteristics** | **Completer**  **(N=45)** | **Non-completer**  **(N=98)** | **P-value** |
| --- | --- | --- | --- |
| **Sex** | F=19 (42.2%)  M=26 (57.8%) | F=54 (55.1%)  M=44 (44.9%) | 0.207 |
| **Age (years)** | 10.7 (3.6) | 12.5 (3.7) | **0.009** |
| **Age at T1D onset (years)** | 5.8 (3.1) | 7.7 (3.7) | **0.002** |
| **Duration of T1D (years)** | 4.3 (3.4) | 4.3 (3.8) | 0.988 |
| **Body weight SDS** | -0.09 (0.92) | 0.02 (1.1) | 0.552 |
| **Body height SDS** | -0.36 (1.05) | -0.32 (1.23) | 0.836 |
| **BMI SDS** | 0.14 (1.18) | 0.18 (0.95) | 0.868 |
| **Treatment modality** | MDI=40 (88.9%)  CSII=5 (11.1%)  NA=0 (0%) | MDI=87 (88.8%)  CSII=6 (6.1%)  NA=5 (5.1%) | 0.338 |
| **CGM status** | Yes=22 (48.9%)  No=22 (48.9%)  NA=1 (2.2%) | Yes=42 (42.9%)  No=52 (53.1%)  NA=4 (4.1%) | 0.587 |
| **HbA1c (mmol/mol)** | 62.3 (20.2) | 64.1 (22.3) | 0.649 |
| **HbA1c (%)** | 7.9 (4.0) | 8.0 (4.2) |  |
| **Time in range** | 61.8 (12.9) | 61.7 (22.2) | 0.981 |
| **Time below range level 1** | 5.0 (3.5) | 4.1 (4.6) | 0.556 |
| **Time below range level 2** | 1.5 (3.8) | 0.8 (1.5) | 0.565 |
| **Time above range level 1** | 23.0 (10.8) | 19.6 (10.9) | 0.452 |
| **Time above range level 2** | 8.4 (6.2) | 13.5 (21.7) | 0.405 |
| **Average glycemia (mmol/l)** | 8.8 (1.6) | 9.4 (3.4) | 0.583 |
| **Average glycemia (mg/dL)** | 158.6 (28.9) | 169.4 (61.3) |  |
| **Coefficient in variation (%)** | 45.5 (9.6) | 36.0 (9.9) | 0.111 |

**ESM Table 4** – Use of CGM and their type throughout the course of the study in study participants (N=143). No data includes study subjects lost to follow-up or not yet reaching the time-point of the visit.

| **CGM type** | **Baseline visit** | **M12** | **M24** | **M36** |
| --- | --- | --- | --- | --- |
| **Freestyle Abbott Libre 1 / 2** | 58 (40.5%) | 76 (53.1%) | 45 (31.5%) | 14 (9.8%) |
| **Medtronic Enlite / Guardian** | 2 (1.4%) | 7 (4.9%) | 16 (11.2%) | 19 (13.3%) |
| **Dexcom G5 / G6 / G7** | 4 (2.8%) | 4 (2.8%) | 6 (4.2%) | 9 (6.3%) |
| **No CGM** | 74 (51.7%) | 5 (3.5%) | 2 (1.4%) | 2 (1.4%) |
| **No data** | 5 (3.5%) | 51 (35.7%) | 74 (51.7%) | 99 (69.2%) |

CGM – continuous glucose monitoring, M12 – month 12, M24 – month 24, M36 – month 36

**ESM Table 5** – Use of CSII/AID and their type throughout the course of the study in study participants (N=143). No data includes study subjects lost to follow-up or not yet reaching the time-point of the visit, or for whom there is no data about their CSII/AID usage.

| **CSII/AID type** | **Baseline visit** | **M12** | **M24** | **M36** |
| --- | --- | --- | --- | --- |
| **Medtronic Minimed 640G** | 6 (4.2%) | 0 (0%) | 0 (0%) | 0 (0%) |
| **Medtronic Minimed 780G** | 0 (0%) | 5 (3.5%) | 15 (10.5%) | 19 (13.3%) |
| **Accu-Check Insight** | 5 (3.5%) | 0 (0%) | 0 (0%) | 0 (0%) |
| **Tandem x:slim t2** | 0 (0%) | 0 (0%) | 5 (3.5%) | 7 (4.9%) |
| **Ypsopump CamAPS** | 0 (0%) | 0 (0%) | 0 (0%) | 2 (1.4%) |
| **No CSII/AID** | 127 (88.8%) | 84 (58.7%) | 48 (33.6%) | 16 (11.2%) |
| **No data** | 5 (3.5%) | 54 (37.8%) | 75 (52.4%) | 99 (69.2%) |

CSII – continuous subcutaneous insulin infusion, AID – automated insulin delivery, M12 – month 12, M24 – month 24, M36 – month 36

**ESM Figure 6** – Mean times in glycemic ranges of the study participants throughout the course of the study. TIR increased from 61.7% (SD 19.3%) to 66.2% (SD 17.0%) (P=0.04).

M12 – month 12, M24 – month 24, M36 – month 36

**ESM Table 7 –** CGM metrics at visits; P-values correspond to the overall F-tests for timepoint in mixed models. Data are shown as mean (SD).

|  | **Baseline visit** | **M12** | **M24** | **M36** | **P-value** | |
| --- | --- | --- | --- | --- | --- | --- |
|  |  |  |  |  | **Crude** | **Covariate-adjusted** |
| **TIR (%)** | 61.7 (19.3)  (N=32) | 58.7 (18.5)  (N=84) | 61.0 (19.7)  (N=67) | 66.2 (17.0)  (N=42) | 0.04 | 0.95 |
| **TBR1 (%)** | 4.4 (4.18)  (N=27) | 4.9 (3.9)  (N=75) | 3.7 (3.1)  (N=67) | 2.5 (2.6)  (N=42) | <0.001 | 0.25 |
| **TBR2 (%)** | 1.0 (2.5)  (N=27) | 0.9 (1.7)  (N=72) | 0.7 (1.3)  (N=64) | 0.6 (1.3)  (N=42) | 0.52 | 0.92 |
| **TAR1 (%)** | 21.0 (10.8)  (N=25) | 21.6 (9.9)  (N=72) | 22.0 (11.6)  (N=67) | 19.3 (6.8)  (N=42) | 0.55 | 0.71 |
| **TAR2 (%)** | 11.4 (17.2)  (N=25) | 13.8 (15.6)  (N=69) | 13.3 (14.0)  (N=64) | 11.1 (14.4)  (N=42) | 0.77 | 0.86 |
| **Average glycemia (mmol/l)** | 9.3 (3.0)  (N=25) | 9.3 (2.5)  (N=73) | 9.3 (2.5)  (N=64) | 9.0 (2.3)  (N=41) | 0.86 | 0.71 |
| **Average glycemia (mg/dL)** | 167.6 (54.1)  (N=25) | 167.6 (45.1)  (N=73) | 167.6 (45.1)  (N=64) | 162.2 (41.5)  (N=41) |  |  |
| **Coefficient of variation (%)** | 41.1 (10.5)  (N=13) | 38.9 (6.3)  (N=47) | 38.9 (7.5)  (N=55) | 37.6 (5.7)  (N=38) | 0.14 | 0.36 |
| **Standard deviation of glycemia (mmol/l)** | 3.6 (1.1)  (N=13) | 3.5 (1.0)  (N=45) | 3.7 (1.4)  (N=55) | 3.3 (0.8)  (N=37) | 0.08 | 0.44 |
| **Standard deviation of glycemia (mg/dL)** | 64.9 (19.9)  (N=13) | 63.1 (18.1)  (N=45) | 66.7 (25.3)  (N=55) | 59.5 (14.5)  (N=37) |  |  |

CGM – continuous glucose monitoring, M12 – month 12, M24 – month 24, M36 – month 36, TIR – time in the target range (3.9–10.0 mmol/L, 70–180 mg/dL), TBR1 – time in level 1 hypoglycemia (3.0–3.8 mmol/L, 54–70 mg/dL), TBR2 – time in level 2 hypoglycemia (<3.0 mmol/L, <54 mg/dL), TAR1 – time in level 1 hyperglycemia (10.1–13.9 mmol/L, 180–‍250 mg/dL), TAR2 – time in level 2 hyperglycemia (>13.9 mmol/L, >250 mg/dL)

**ESM Table 8 –** CGM metrics at visits stratified by the type of therapy (MDI vs. CSII/AID), P-values correspond to the test of slope being zero. Data are shown as mean (SD).

|  | **Baseline visit** | | **M12** | | **M24** | | **M36** | | **P-value** |
| --- | --- | --- | --- | --- | --- | --- | --- | --- | --- |
| **Type of therapy** | **MDI (N=127)** | **CSII/AID (N=11)** | **MDI**  **(N=85)** | **CSII/AID**  **(N=7)** | **MDI**  **(N=47)** | **CSII/AID**  **(N=22)** | **MDI**  **(N=16)** | **CSII/AID**  **(N=28)** |  |
| **Average glycemia (mmol/l)** | 9.4  (3.1)  (N=22) | 9.3  (3.0)  (N=2) | 9.3  (2.6)  (N=67) | 8.7  (1.3)  (N=6) | 9.9  (2.8)  (N=42) | 8.3  (0.9)  (N=22) | 10.4  (3.5)  (N=13) | 8.3  (0.9)  (N=28) | 0.71 |
| **Average glycemia (mg/dL)** | 169.4  (55.9)  (N=22) | 167.6  (54.1)  (N=2) | 167.6  (46.9)  (N=67) | 156.8  (23.5)  (N=6) | 178.4  (50.5)  (N=42) | 149.6  (16.3)  (N=22) | 187.4  (63.1)  (N=13) | 149.6  (16.3)  (N=28) |  |
| **Coefficient of variation (%)** | 41.6  (9.3)  (N=11) | 38.5  (21.4)  (N=2) | 38.7  (6.4)  (N=44) | 41.5  (6.2)  (N=3) | 40.5  (7.8)  (N=35) | 36.1  (6.4)  (N=20) | 39.2  (7.8)  (N=10) | 37.1  (4.8)  (N=28) | 0.36 |
| **Standard deviation of glycemia (mmol/l)** | 3.7  (0.9)  (N=11) | 3.2  (2.2)  (N=2) | 3.5  (1.0)  (N=42) | 4.0  (0.4)  (N=3) | 4.0  (1.5)  (N=35) | 3.1  (0.8)  (N=20) | 3.9  (1.2)  (N=9) | 3.1  (0.6)  (N=28) | 0.44 |
| **Standard deviation of glycemia (mg/dL)** | 66.7  (16.3)  (N=11) | 57.7  (39.7)  (N=2) | 63.1  (18.1)  (N=42) | 72.1  (7.3)  (N=3) | 72.1  (27.1)  (N=35) | 55.9  (14.5)  (N=20) | 70.3  (21.7)  (N=9) | 55.9  (10.9)  (N=28) |  |

CGM – continuous glucose monitoring, M12 – month 12, M24 – month 24, M36 – month 36

**ESM Figure 9** – Mean times in glycemic ranges of the study participants (Case) and their matched controls from Czech National Pediatric Diabetes Registry (Control) at three years. We observed no significant differences in any of the ranges.
